# The Use of Copper Oxides as Cross-Linking Substances for Chloroprene Rubber and Study of the Vulcanizates Properties. Part I

**DOI:** 10.3390/ma14195535

**Published:** 2021-09-24

**Authors:** Piotr Kobędza, Aleksandra Smejda-Krzewicka, Krzysztof Strzelec

**Affiliations:** Faculty of Chemistry, Institute of Polymer and Dye Technology, Lodz University of Technology, Stefanowskiego 16, 90-537 Łódź, Poland; aleksandra.smejda-krzewicka@p.lodz.pl (A.S.-K.); krzysztof.strzelec@p.lodz.pl (K.S.)

**Keywords:** chloroprene rubber, copper(I) oxide, copper(II) oxide, cross-linking, IR, DSC, TGA, mechanical properties

## Abstract

The purpose of this work was to verify the ability to cross-link the chloroprene rubber (CR) by using copper oxides: copper(I) oxide or copper(II) oxide. The use of copper oxides arises from the need to limit the application of ZnO as a cross-linking agent of CR. The obtained results indicate that CR compositions cross-linked with copper oxides are characterized by good mechanical properties and a high cross-linking degree. The results show that the type and the amount of copper oxides influence the cross-linking of the CR and the properties of the vulcanizates. For compositions containing copper(II) oxide, the properties are linearly dependent on the amount of CuO. Such a relationship is difficult to notice in the case of the use of copper(I) oxide—when analyzing individual parameters, the best results are obtained for different samples. Infrared spectroscopy (IR) studies confirmed the possibility of cross-linking of chloroprene rubber with copper oxides. This is evidenced by the characteristic changes in the intensity of the bands. Structural changes in the material during heating were determined by the thermal analysis—differential scanning calorimetry (DSC) and thermogravimetric analysis (TGA). Regardless of the type and amount of copper oxide, all compositions exhibit similar characteristics, and there are no significant changes in the glass transition temperature of the material.

## 1. Introduction

Metal oxides are inorganic compounds widely used in polymer processing technology. They can be used as cross-linking agents, activators, and fillers, as well as desiccants or pigments, in amounts usually several phr. Due to their characteristics, metal oxides usually fulfill several functions simultaneously, e.g., a filler and colorant [1,2,3].

The most important component in the rubber mixture, next to the rubber itself, is the cross-linking substance. Under the influence of the cross-linking substance, a network is formed through the bonds connecting the chains of elastomers. In elastomer technology, metal oxides are used to cross-link materials containing atoms belonging to the halogen group in their main chains. This rubber group includes chloroprene rubber [4,5,6,7,8], chlorosulfonated polyethylene [9] and halogenobutyl rubbers: chlorobutyl [10,11,12] and bromobutyl [12,13]. For cross-linking of these materials, a mixture of two oxides is used: zinc oxide [4,5,6,7,8,9,10,11,12,13,14,15,16] and magnesium oxide [4,6,7,8,9,11,13,14,16,17,18]. Zinc oxide is a compound in the form of white hexagonal crystals. Magnesium oxide is in the form of a white, finely divided powder. Cross-linking of rubber containing halogen atoms occurs as a result of the reaction of this atom with zinc oxide, resulting in the formation of a Lewis acid (e.g., zinc chloride) [5,6,7,10,15]., whereas magnesium oxide is an acceptor of the formed hydrogen chloride [15]. Additionally, ZnO and MgO can be used for the cross-linking of carboxylated acrylonitrile-butadiene rubber [16,17,18].

Substances not commonly used up till now are copper oxides and iron oxides. Copper(I) oxide is in the form of a red-brown powder and occurs in the natural environment as cuprite. Copper(II) oxide is in the form of a black powder. Iron(III) oxide, in the form of a brown powder, commonly occurs as rust, while, in nature, it can be found as hematite. However, iron(II,III) oxide, in the form of a black powder, exists as magnetite. Copper oxides and iron oxides can substitute zinc oxide and magnesium oxide in the cross-linking of chloroprene rubber, chlorosulfonated polyethylene, or chlorobutyl rubber. Copper oxides and iron oxides are also effective in cross-linking CR and chlorosulfonated polyethylene (CSM) mixtures with styrene-butadiene rubber and butadiene rubber [19,20,21,22,23,24,25,26,27].

Another important function of metal oxides in rubber mixtures is their filling. The main function of the fillers is their influence on the processing properties of the mixtures, as well as providing appropriate mechanical and functional (physical and chemical) properties of rubber products. However, in the technology of elastomers, metal oxides are more often used as a component of another (mineral) filler than separately. Aluminum(III) oxide in its pure form is not used as a filler, but it is an important building block of aluminosilicates used in polymer technology, namely kaolin. Kaolin, a product of natural weathering of igneous rocks, contains kaolinite, i.e., phyllosilicate, as the main ingredient. Kaolin occurs in the form of a white or cream powder. Kaolin is used as a cheap semi-active filler but also causes blistering of the vulcanizate [28,29,30,31]. Calcium oxide is also used as a filler [32]. CaO is a white powder, obtained by burning limestone. ZnO also exhibits vulcanizate strengthening properties. However, both CaO and ZnO are at most semi-reactive fillers and are, therefore, rarely used as the primary filler [32]. More often, the incorporation of metal oxide in another function additionally strengthens the entire material.

Activators are used to increase the effectiveness of the accelerator. There are two main types of activators: divalent metal oxides and fatty acids. Often, both types of activators are used simultaneously, with metal oxides being used in greater amounts. Usually, it is 4–6 parts by weight of oxide with 0.5–1.5 parts. wt. acid. As an activator can be used previously mentioned in other functions ZnO, MgO, or CaO [33].

Any metal oxide incorporated into the rubber mixture will give it its color; therefore, they can be classified as coloring substances. The most commonly used metal oxide pigment is titanium(IV) oxide [34,35]. TiO_2_ forms white crystals in three naturally occurring polymorphs: anatase and rutile, crystallizing in the tetragonal system, and brookite forming crystals in the orthorhombic system. Anatase and rutile are common minerals, while brookite belongs to the group of a very rare minerals. TiO_2_ has one of the purest shades of white; however, it is an expensive material. A cheaper substitute for TiO_2_ is ZnO [36].

The purpose of this work was to verify the ability to cross-link the chloroprene rubber (CR) by using copper oxides: copper(I) oxide or copper(II) oxide. Seeking a substitute for ZnO as a cross-linking agent results from the restrictions on its use implemented by the European Union. As an alternative to ZnO, different metal oxides can be used as a cross-linking agent. The choice of copper oxides results from the ability to cross-link CR blends with styrene-butadiene rubber (SBR) or butadiene rubber (BR) [21,22,23,24,25,26,27]. The advantage of using copper oxides is the smaller amount needed for cross-linking elastomer blends containing CR compared to other cross-linkers. A smaller amount of used materials reduces the cost of making the composition.

## 2. Experimental Part

### 2.1. Materials

In this study, chloroprene rubber (Baypren^®^216 MV from Lanxess GmbH, Cologne, Germany), with a density of 1.23 g/cm^3^ and Mooney viscosity (ML 1 + 4 100 °C) of 43 ± 5, was used. As a cross-linking agent, two copper oxides were used: copper(I) oxide (POCH S.A., Gliwice, Poland) with a density of 6.00 g/cm^3^, pureness >99%, and particle size ≤7 µm; and copper(II) oxide (Sigma-Aldrich Chemie GmbH, Steinheim am Albuch, Germany) with a density 6.32 g/cm^3^, pureness 98%, and particle size <10 µm. For comparison, a composition containing the standard chloroprene rubber cross-linking system was made. For this purpose, zinc oxide (Pharma Cosmetic, Cracow, Poland) with a density of 5.47 g/cm^3^ and pureness >99%, and magnesium oxide (PPH Galfarm Sp. z o. o., Cracow, Poland) with a density of 3.58 g/cm^3^; were used. Stearic acid (Chemical Worldwide Business Sp. z o.o., Słupca, Poland) with a density of 0.85 g/cm^3^ was used as a dispersing agent.

### 2.2. Research Methods

The chloroprene rubber composites were prepared using a Krupp-Gruson laboratory two-roll mill (Laborwalzwerk 200x450, Krupp-Gruson, Magdeburg-Buckau, Germany) with a roll diameter of 200 mm and a length of 450 mm. The temperature of the roll was 20–25 °C, while the speed of the front roll was 200 rpm, with the roll’s friction of 1:1.25. The preparation of one composition lasted 10 min. Then, the material was conditioned for 24 h. At the beginning, rubber was incorporated into two-roll mill to plasticize it. Stearic acid was then added to facilitate the insertion of the oxides. Finally, the metal oxides were incorporated: copper(I) oxide, copper(II) oxide, or a mixture of magnesium oxide and zinc oxide (in the given order).

Vulcametric measurements were determined by the Alpha Technologies MDR 2000 rotorless rheometer (MDR 2000, Alpha Technologies, Hudson, OH, USA), heated to 160 °C. The oscillation frequency was 1.67 Hz. The test was 60 min and performed according to ASTM D5289 [37]. The torque increment after a given time of heating was calculated from Formula (1):

(1) $$\Delta M_{x}=M_{x}-M_{\min}.$$

Vulcanization was performed in an electrically heated hydraulic press. Appropriate amounts of the compositions were placed in steel molds, which were placed in a press at a temperature of 160 °C and a pressure of 200 bar. The vulcanization time was 45 min.

The determination of equilibrium volume swelling was performed. Samples were cut from the prepared vulcanizates in four different shapes. Each of them weighed from 25 to 50 mg, with an accuracy of 0.1 mg. Then, the samples were placed with solvents: toluene or heptane, in a weighing bottle. Prepared samples were placed in a thermostatic chamber for 72 h at 25 ± 1 °C, which, after this time, was bathed with diethyl ether, dried on filter paper, and then weighed again. Then, the samples were dried in a dryer at the temperature of 50 °C to a constant weight, and they were reweighed. The equilibrium volume swelling was calculated from Formula (2):

(2) $$Q_{V}=Q_{W}\cdot \frac{d_{v}}{d_{s}}.$$

The equilibrium weight swelling was calculated from Formula (3):

(3) $$Q_{w}=\frac{m_{s}-m_{d}}{m_{d}^{*}}.$$

The reduced sample weight was calculated from Formula (4):

(4) $$m_{d}^{*}=m_{d}-m_{0}\cdot \frac{m_{m}}{m_{t}}.$$

Determination of Mooney-Rivlin elasticity constants was performed. The elasticity constants were calculated based on the Mooney-Rivlin Equation (5) [38,39]:

(5) $$\frac{P}{2A_{0}\cdot \left(\lambda -\lambda^{-2}\right)}=C_{1}+C_{2}\cdot \lambda^{-1}.$$

Extraction of vulcanizates in the boiling acetone vapors in a Soxhlet apparatus for 48 h was performed. After the given time, the samples were dried to a constant weight in a vacuum oven at 50 °C. Results of the extraction allowed to determine the content of non-rubber substances. The value of real extract was calculated from Formula (6):

(6) $$E_{R}=\frac{m_{0}-m}{m_{0}}.$$

Mechanical properties: stress at elongation 100%, 200%, 300%, tensile strength, and elongation at break were tested by the universal testing machine ZwickRoell 1435 (1435, ZwickRoell, Ulm, Germany). The tests were performed according to PN-ISO 37:2007 [40].

Infrared spectra were made using the FTIR-ATR method and recorded using a Thermo Scientific Nicolet 6700 FTIR spectrometer (Nicolet 6700 FT-IR Spectrometer, Thermo Fisher Scientific, Waltham, MA, USA). Samples for infrared tests were prepared from elastomer blends before and after their cross-linking.

Thermal analysis—TGA and DSC—was performed using a Mettler Toledo TGA/DSC 1 device (TGA/DSC 1, Mettler-Toledo, Columbus, OH, USA). TGA analyses were performed using a two-step procedure. First, samples of vulcanizates were heated in the temperature range of 25–600 °C in an argon atmosphere (flow rate 50 mL/min), with a heating rate of 20 °C/min Next, the gas was changed into the air (flow rate 50 mL/min), and the heating was continued up to 900 °C, with the same heating rate. DSC measurements were performed on rubbers blends. Samples were heated from −100 °C to 250 °C, with a heating rate of 10 °C/min. Nitrogen (80 mL/min) was used as the protective gas, whereas liquid nitrogen was applied to cool the sample before the measurement.

## 3. Results and Discussion

To investigate the ability of chloroprene rubber cross-linking with copper oxides, compositions containing 1, 2, 3, 4, or 5 weight parts of copper oxide/100 weight parts of CR (phr), were prepared (Table 1). The following oxides were used: copper(I) oxide (Cu_2_O) or copper(II) oxide (CuO). For comparative purposes, CR cross-linked with a standard cross-linking system, i.e., a mixture of zinc oxide (5 phr of ZnO) and magnesium oxide (4 phr of MgO), was also prepared. In addition, CR was examined with regards to susceptibility to thermal cross-linking by preparing a composition containing only chloroprene rubber. The purpose of copper oxides use is to obtain vulcanizates with better properties compared to the materials obtained with the use of a standard cross-linking system. In addition, the use of zinc oxide is limited and alternatives should be sought.

### 3.1. Vulcametric Parameters of CR Compositions Containing Copper Oxides

**Figure 1:**
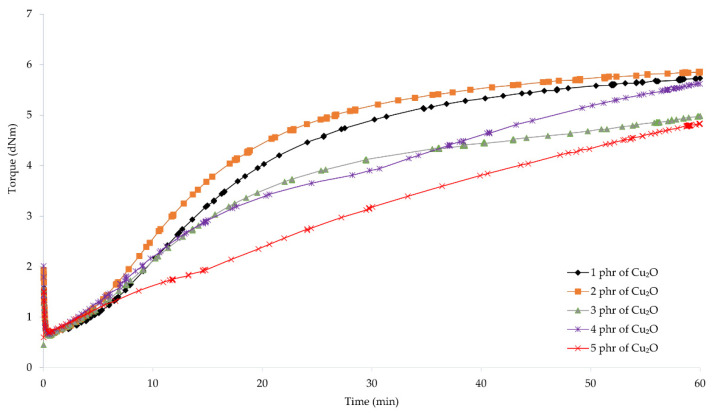
Vulcametric kinetics of chloroprene rubber cross-linked with copper(I) oxide (1–5 phr of Cu_2_O).

**Figure 2:**
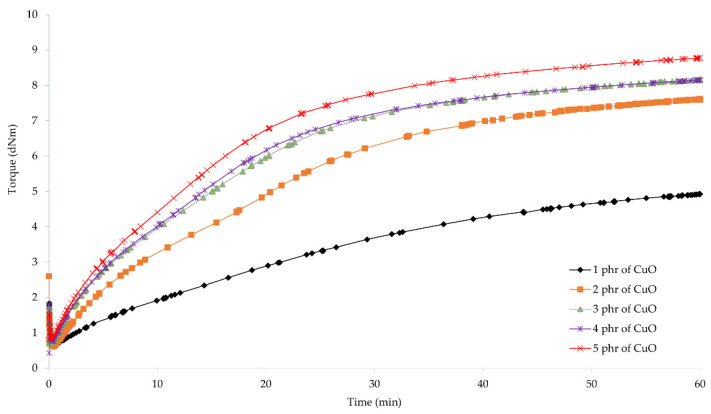
Vulcametric kinetics of chloroprene rubber cross-linked with copper(II) oxide (1–5 phr of CuO).

To determine the possibility of cross-linking chloroprene rubber with copper oxides and the characteristics of the vulcanization course, vulcametric parameters were determined. The results showed that the compositions containing copper(I) oxide were characterized by scorch time in the range from 2.7 (for the CR/Cu_2_O-2) to 7.4 min (for the CR/Cu_2_O-5) (Table 2, Figure 1). For compositions containing copper(II) oxide, the *t*_02_ values were from 1.7 (for the CR/CuO-4) to 8.1 min (for the CR/CuO-1) (Table 2, Figure 2). For comparison, the scorch time of the CR compound containing the conventional cross-linking system was equal to 4.5 min. However, for CR without any metal oxide, *t*_02_ was 6.4 min.

The shortest vulcanization time was obtained for the CR/ZnO/MgO, equal to 27.5 min. Replacing zinc oxide and magnesium oxide with copper oxides results in a longer vulcanization time. For the CR/Cu_2_O-1, *t*_90_ = 38.8 min. As the content of Cu_2_O in the compound increases, the vulcanization time lengthens, reaching 53.7 min for the CR/Cu_2_O-5. In the case of compositions containing CuO, the shortest vulcanization time was obtained for the CR/CuO-4 (*t*_90_ = 38.6 min), while the longest vulcanization time was achieved for the CR/CuO-3 (*t*_90_ = 44.9 min). For the CR without any metal oxide, *t*_90_ = 49.0 min.

The minimum torque for the compounds containing 3–5 phr of Cu_2_O and 5 phr of CuO was equal to 0.59 dN · m. The same value was obtained for the composition containing zinc oxide and magnesium oxide. For the CR/Cu_2_O-1, the minimum torque was the smallest and equal to 0.53 dN · m, whereas the minimum torque was the greatest for the CR/CuO-4 (*M*_min_ = 0.62 dN · m). For CR without metal oxide, *M*_min_ = 0.55 dN · m. These results indicate that all prepared blends had similar viscosity.

Torque increment after 45 min of heating of the CR/Cu_2_O-5 was the largest and equal to 3.91 dN · m. With the decreasing amount of Cu_2_O in the compounds, the value of the torque increment also decreased, reaching the value of 2.63 dN · m for the CR/Cu_2_O-1. For the compositions containing copper(II) oxide, the greatest value of torque increment after 45 min of heating was obtained for the CR/CuO-5 (Δ*M*_45_ = 2.65 dN · m). For the application of the copper(II) oxide, with the decreasing amount of CuO in the composition, the torque increment also decreases, as in the case of Cu_2_O. For the CR/CuO-1, Δ*M*_45_ = 0.88 dN · m. The CR without any metal oxide obtained the value of Δ*M*_45_ = 1.86 dN · m, while, for the CR containing the standard cross-linking system, Δ*M*_45_ = 3.77 dN · m.

The obtained results show that, with an increasing amount of copper oxides in the chloroprene rubber, the torque increment also increases. Compounds containing copper(I) oxide achieve higher values of the torque increment than compositions containing copper(II) oxide. For the CR/Cu_2_O-1, CR/CuO-4, and CR/CuO-5, the values of the torque increment were averagely 2.64 dN · m. This indicates that copper(I) oxide is more effective in creating bonds between the chains of chloroprene rubber—the minimum amount of Cu_2_O is as effective in cross-linking as larger amounts of copper(II) oxide (4–5 phr of CuO). This may indicate the formation of a more complex network structure in the case of using Cu_2_O. The torque after heating the CR/Cu_2_O-5 for 45 min is greater than for the CR/ZnO/MgO. The scorch time of the compositions containing 1–3 phr of Cu_2_O or at least 2 phr of CuO is shorter than for the CR/ZnO/MgO. The incorporation of copper oxide, regardless of its type or amount, causes a longer vulcanization time than in the case of zinc oxide and magnesium oxide. Such results indicate a faster start of cross-linking of the composition, simultaneously extending the entire process. This may arise from the need to supply more energy needed to efficiently use all the incorporated copper oxide.

### 3.2. Equilibrium Volume Swelling of CR Cross-Linked with Copper Oxides

Obtained results of equilibrium volume swelling confirmed the conclusions of the analysis of the vulcametric kinetics that the presence of copper oxide causes cross-linking of chloroprene rubber. The highest value of equilibrium volume swelling in toluene (*Q_V_*^T^ = 19.26 cm^3^/cm^3^) was obtained for thermo-cross-linked vulcanizates (Table 3). CR cross-linked with CuO achieved lower *Q_V_*^T^ values than CR cross-linked with Cu_2_O. The lowest value of equilibrium volume swelling in toluene (*Q_V_*^T^ = 4.68 cm^3^/cm^3^) was achieved for the CR/CuO-5. Very similar results were obtained for vulcanizates containing 3 and 4 phr of CuO. In the case of the use of CuO as the cross-linking agent, the highest value was obtained for the CR/CuO-1, for which *Q_V_*^T^ = 8.39 cm^3^/cm^3^. This value is still lower than the lowest value of the equilibrium volume swelling in toluene for vulcanizates containing copper(I) oxide (*Q_V_*^T^ = 8.50 cm^3^/cm^3^ for the CR/Cu_2_O-1). In turn, the highest *Q_V_*^T^ value, equal to 10.56 cm^3^/cm^3^, was achieved for the CR/Cu_2_O-2. For comparison, the *Q_V_*^T^ value for the CR/ZnO/MgO was equal to 12.24 cm^3^/cm^3^.

In the case of equilibrium volume swelling in heptane, the values are comparable for all vulcanizates containing copper oxide. For vulcanizates containing copper(I) oxide, the *Q_V_*^H^ values ranged from 0.37 cm^3^/cm^3^ (for the CR/Cu_2_O-4) to 0.51 cm^3^/cm^3^ (for the CR/Cu_2_O-1). For vulcanizates containing copper(II) oxide, the lowest equilibrium volume swelling in heptane was obtained for the CR/CuO-1 (*Q_V_*^H^ = 0.38 cm^3^/cm^3^), while the highest value was achieved for the CR/CuO-5 (*Q_V_*^H^ = 0.48 cm^3^/cm^3^). For comparison, *Q_V_*^H^ = 0.39 cm^3^/cm^3^ for thermo-cross-linked CR, while, for vulcanizate containing ZnO and MgO, *Q_V_*^H^ = 0.45 cm^3^/cm^3^.

The results of the equilibrium swelling measurements show that CR cross-linked with copper(II) oxide exhibits greater resistance to solvents. This proves a good cross-linking degree of these vulcanizates. It can also be observed that, with the increasing amount of CuO in the vulcanizate, the *Q_V_*^T^ value decreases; thus, the cross-linking degree increases, reaching almost identical values for the content of 3–5 phr of CuO. This proves the formation of an increasingly complex network structure along with the increasing amount of CuO in the composition. Moreover, the use of larger amounts of copper(II) oxide does not proportionally affect the expansion of the network. A similar relationship is not maintained for CR cross-linked with Cu_2_O. The lowest *Q_V_*^T^ value was obtained for the CR/Cu_2_O-1, and the highest for the CR/Cu_2_O-2. However, as the content of copper(I) oxide exceeds 2 phr, the *Q_V_*^T^ value decreases, reaching 9.76 cm^3^/cm^3^ for the CR/Cu_2_O-5. Such results may indicate the agglomeration of Cu_2_O and, consequently, a reduction in the cross-linking efficiency of the composition. Importantly, the use of copper oxides results in a *Q_V_*^T^ value lower than in the case of conventionally cross-linked CR (with ZnO and MgO), which proves the effectiveness of Cu_2_O and CuO as the cross-linking agent, forming a complex network structure.

### 3.3. Analysis of the Cross-Linking Degree by Determining the Elasticity Constants

The elasticity constants calculated from the Mooney-Rivlin equation allow the determination of the network formed during the cross-linking. The first elasticity constant is related to the cross-linking degree—the greater the constant value, the greater the vulcanizate cross-linking degree is. The second elasticity constant can be equated with the deviation of the obtained network from the ideal network. For vulcanizates containing Cu_2_O, the lowest value of *C*_1_ was equal to 0.47 kG/cm^2^ (for the CR/Cu_2_O-1), while the highest value was equal to 0.67 kG/cm^2^ (for the CR/Cu_2_O-3) (Table 3). The value of the second elasticity constant was also the lowest for the CR/Cu_2_O-1 (*C*_2_ = 0.50 kG/cm^2^), while the highest for the CR/Cu_2_O-5 (*C*_2_ = 2.44 kG/cm^2^). For vulcanizates containing CuO, the lowest value of *C*_1_ was equal to 0.38 kG/cm^2^ (for the CR/CuO-1), while the highest was equal to 1.32 kG/cm^2^ (for the CR/CuO-5). In the case of *C*_2_, the lowest value was recorded for the CR/CuO-5 (*C*_2_ = 1.36 kG/cm^2^), and the highest for the CR/CuO-2 (*C*_2_ = 3.70 kG/cm^2^). For comparison, the thermo-cross-linked CR vulcanizate obtained the lowest value of the first elasticity constant among all the tested samples (*C*_1_ = 0.34 kG/cm^2^). The value of *C*_2_ was equal to 2.22 kG/cm^2^. In turn, the vulcanizate containing ZnO and MgO was characterized by the value of *C*_1_ equal to 0.58 kG/cm^2^. This is a lower value of the *C*_1_ constant than for the vulcanizates containing at least 2 phr of CuO or Cu_2_O (except for the CR/Cu_2_O-4, for which *C*_l_ = 0.55 kG/cm^2^). The value of the *C*_2_ constant was the smallest among all tested samples (*C*_2_ = 0.46 kG/cm^2^).

The analysis of the elasticity constants results confirms the possibility of CR cross-linking with copper oxides. Higher values of the first elasticity constant, equated to an increase in the cross-linking degree, were obtained for vulcanizates containing CuO. The use of 2 phr of CuO results in a value of *C*_1_ comparable to the use of 2–3 phr of Cu_2_O. However, the incorporation of different amounts of CuO results in a linear relationship—as the amount of CuO increases, the value of *C*_1_ also increases. Such a relationship does not occur while applicating Cu_2_O. In this case, the CR/Cu_2_O-1 has the lowest value of *C*_1_, and the CR/Cu_2_O-3 has the highest value. It is noteworthy that the first elasticity constant for conventionally cross-linked CR was lower than for most vulcanizates containing copper oxides. The exceptions were samples containing 1 phr of Cu_2_O or CuO and 4 phr of Cu_2_O. These results confirm the observations from the measurements of the equilibrium volume swelling. The emerging network structure is more complex when more CuO is incorporated, whereas, when Cu_2_O is used, agglomeration may occur, which hinders the effective use of the oxide.

### 3.4. Real Extract of CR Cross-Linked with Copper Oxides

To confirm that cross-linking of chloroprene rubber with copper oxides leads to the network formation, the cross-linked samples were subjected to exhaustive extraction in the vapor of boiling acetone, which elutes non-rubber components and the gel fraction of CR. The real extract reaches lower values for the samples cross-linked to a greater degree. In the case of CR cross-linked with Cu_2_O, the real extract values ranged from 0.084 mg/mg (for the CR/Cu_2_O-3 and CR/Cu_2_O-5) to 0.102 mg/mg (for the CR/Cu_2_O-2) (Table 3). In the case of vulcanizates containing CuO, the value of the real extract decreased with increasing content of copper(II) oxide, reaching the highest value for the CR/CuO-1 (*E*_R_ = 0.090 mg/mg), and the lowest for the CR/CuO-5 (*E*_R_ = 0.057 mg/mg). For comparison, the thermo-cross-linked CR reached *E*_R_ = 0.090 mg/mg. However, for the CR/ZnO/MgO, *E*_R_ = 0.159 mg/mg, which was the highest value among all tested samples.

The analysis of the real extract results shows that the amount and type of copper oxide affect the cross-linking of chloroprene rubber. The use of CuO as a cross-linking substance is more effective than Cu_2_O. As the CuO content in the vulcanizate increases, the *E*_R_ value decreases. When Cu_2_O was used, the highest *E*_R_ value was obtained for the CR/Cu_2_O-2, and the lowest value was achieved for the samples containing 3 and 5 phr of Cu_2_O. The real extract results confirm the hypothesis of Cu_2_O agglomeration, which hinders cross-linking of CR, and the emerging network proportionally complexed to the amount of CuO used. It is important that the real extract values for vulcanizates containing copper oxides are noticeably lower than for the conventionally cross-linked CR sample (with ZnO and MgO).

### 3.5. Mechanical Properties of CR Cross-Linked with Copper Oxides

**Figure 3:**
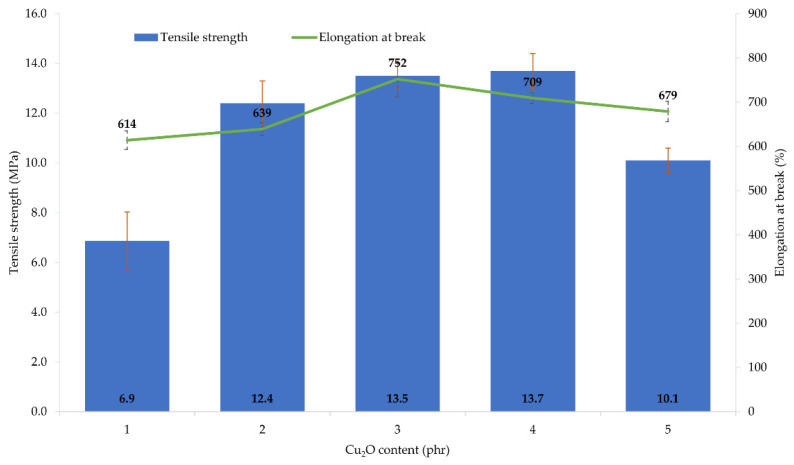
Mechanical properties of the CR compositions containing copper(I) oxide (1–5 phr of Cu_2_O); *T* = 160 °C, *t* = 45 min.

**Figure 4:**
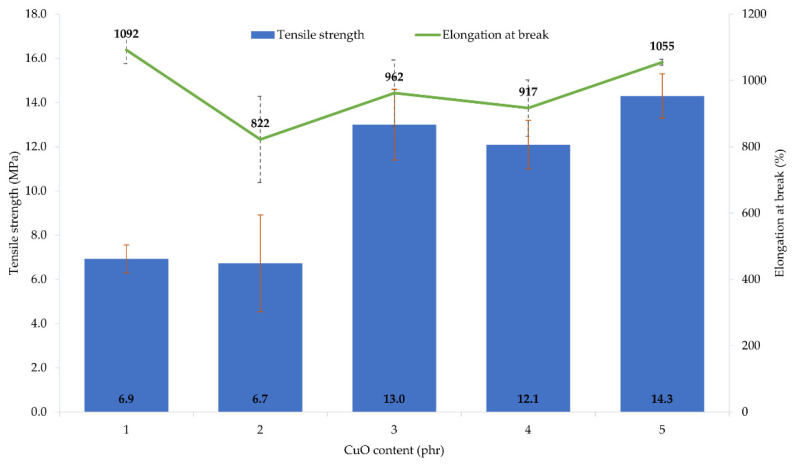
Mechanical properties of the CR compositions containing copper(II) oxide (1–5 phr of CuO); *T* = 160 °C, *t* = 45 min.

The performed tests showed that the amount and type of copper oxide affect the mechanical properties of CR vulcanizates. The thermo-cross-linked CR sample obtained the tensile strength value equal to 1.9 MPa, which was the lowest value among all tested samples. For the CR/Cu_2_O-1, *TS*_b_ = 6.9 MPa (Table 4, Figure 3), whereas the incorporation of at least 2 phr of Cu_2_O results in increasing the vulcanizates tensile strength to values in the range from *TS*_b_ = 10.1 MPa for the CR/Cu_2_O-5, to *TS*_b_ = 13.7 MPa for the CR/Cu_2_O-4. A similar dependence can be observed for vulcanizates containing CuO. The incorporation of 1 or 2 phr of CuO causes a tensile strength value of less than 7 MPa (Table 4, Figure 4), whereas, incorporation of at least 3 phr of CuO leads to increasing the *TS*_b_ to at least 12.1 MPa (for the CR/CuO-4), up to 14.3 MPa (for the CR/CuO-5). For comparison, CR cross-linked with ZnO and MgO was characterized by *TS*_b_ = 7.8 MPa, which is slightly higher than the tensile strength values of vulcanizates containing small amounts of copper oxides.

The elongation at break of the thermo-cross-linked CR, standard cross-linked (with ZnO and MgO) or cross-linked with copper(I) oxide, was comparable. The thermo-cross-linked CR reached the value of *E*_b_ = 779%, while the CR cross-linked with ZnO and MgO obtained the value of *E*_b_ = 680%. However, the elongation at break values for the CR cross-linked with copper(I) oxide ranged from 614% (for the CR/Cu_2_O-1) to 752% (for the CR/Cu_2_O-3). In turn, the values of elongation at break for vulcanizates containing CuO are noticeably higher. They ranged from 822% (for the CR/CuO-2) to 1092% (for the CR/CuO-1).

The amount and type of copper oxide affect the mechanical properties of the prepared vulcanizates. The use of at least 2 phr of Cu_2_O or 3 phr of CuO results in obtaining vulcanizates with the tensile strength above 10 MPa, while the vulcanizate containing the standard cross-linking system was characterized by the tensile strength of 8 MPa. These results indicate that the mechanical strength does not unequivocally correlate with the degree of cross-linking of the composition. This may indicate the additional function of the copper oxides to strengthen the composition. Analysis of the elongation at break result showed that the values for the sample containing ZnO and MgO and the samples containing Cu_2_O were comparable, whereas, for vulcanizates containing CuO, the elongation increased from 20% to even 55% compared to the reference sample.

### 3.6. Infrared Spectra of CR Cross-Linked with Copper Oxides

**Figure 5:**
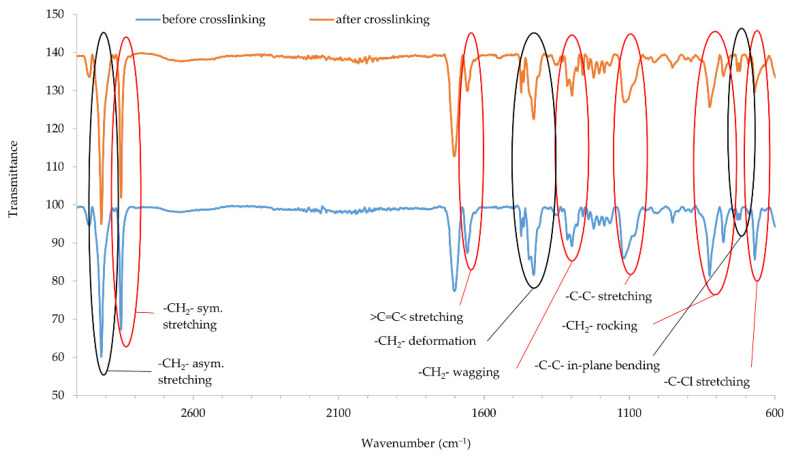
IR spectra of CR composition cross-linked with copper(I) oxide (3 phr of Cu_2_O); *T* = 160 °C, *t* = 45 min.

**Figure 6:**
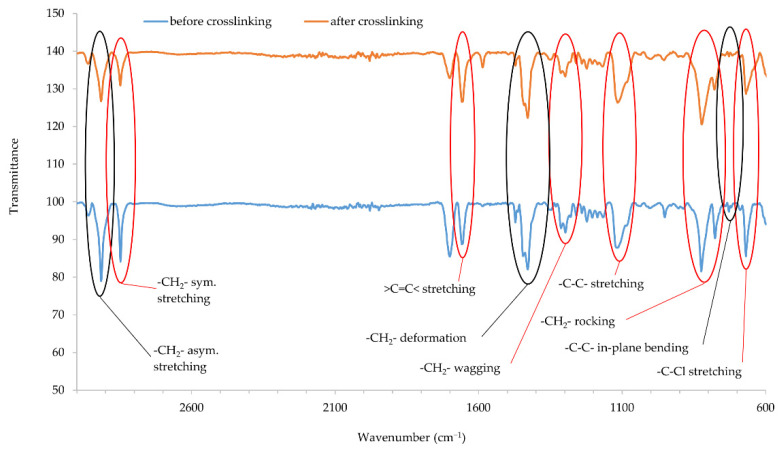
IR spectra of CR composition cross-linked with copper(II) oxide (3 phr of CuO); *T* = 160 °C, *t* = 45 min.

Infrared spectra confirmed that copper oxides can cross-link the CR compositions. An increase in the intensity of the peaks at the wavenumbers of 2915 and 2847 cm^−1^ corresponds with the stretching of -CH_2_-, asymmetric and symmetric, respectively (Figure 5 and Figure 6). The decreasing intensity of the absorption peak at the wavenumber of 1655 cm^−1^ (>C=C< stretching) may indicate the formation of bonds between elastomer macromolecules, by breaking the double bonds in the CR chain. Medium and strong peaks at the wavenumbers 1471 and 1428 cm^−1^, respectively, are attributed to -CH_2_- deformations. Shifts in the intensity of these bands indicate the changes taking place in CR during its heating in the presence of copper oxides. Changing intensity of the absorption peak at the wavenumber of 1296 cm^−1^ corresponds with the wagging of -CH_2_-. In turn, the change in band intensity at wavenumber 1109 is attributed to the C-C stretch of the CR main chain, which can provide the reorganization of structures when the blends were heated in the presence of copper oxides. The decreasing intensity of the absorption peak at the wavenumber of 822 cm^−1^ is attributed to -CH_2_- rocking corresponds with the changes in methylene groups shown in other bands. The absorption peak at the wavenumber of 727 cm^−1^ is attributed to in-plane bending of -C-C- bonds in CR main chain. The decreasing intensity of the absorption peak at the wavenumber of 668 cm^−1^ (-C-Cl stretching) clearly illustrates that, when the CR is heated in the presence of copper oxides, the elastomer cross-links.

IR spectra analysis allows the assumption that copper oxides can be a substitute for zinc oxide as cross-linking substance. Instead of the Lewis acid ZnCl_2_ formed during traditional CR cross-linking, it is likely that CuCl or CuCl_2_ is formed when a given copper oxide is used (Cu_2_O and CuO, respectively). The type of used copper oxide does not significantly change the intensity of the bands, which proves the formation of a comparable network structure.

### 3.7. Thermal Analysis of CR Cross-Linked with Copper Oxides

**Figure 7:**
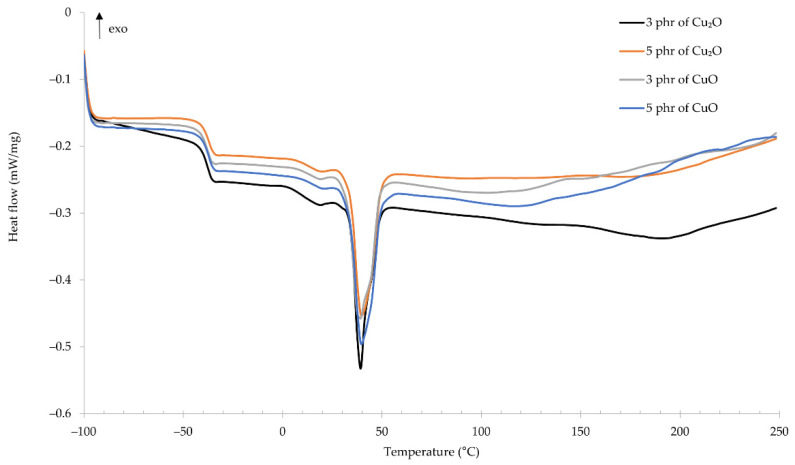
DSC spectrum of CR compositions; *T* = 160 °C, *t* = 45 min.

Using the differential scanning calorimetry allowed us to determine the glass transition temperature of the compositions. For all tested samples, the glass transition temperature was approximately −38 °C (Figure 7). For the CR/Cu_2_O-3: *T*_g_ = −38.51 °C, for the CR/Cu_2_O-5: *T*_g_ = −38.15 °C, for the CR/CuO-3: *T*_g_ = −38.72 °C, and for the CR/Cu_2_O-3: *T*_g_ = −37.95 °C. Almost identical glass transition temperatures prove that the type and amount of copper oxide do not influence this parameter. For comparison, standard cross-linked CR has a glass transition temperature ranging from −50 to −40 °C [41,42]. This proves a slight influence of copper oxides on increasing the glass transition temperature of the CR composition. At the temperature of ~39 °C, a significant endothermic peak appears, which proves the melting of the crystalline structures formed over time in chloroprene rubber during its storage. Up to the temperature of ~100 °C, the characteristics of the curves are almost identical for all tested samples. Above this temperature, the characteristics fluctuate, which indicates changes in the material depending on its composition. In the temperature range from 125 to 179 °C, an exothermic peak appears, which proves that the composition is cross-linked by copper oxides. In the case of samples containing copper(I) oxide, the exothermic peak is wider. For the CR/Cu_2_O-3, the peak is in the range of 126–179 °C with a maximum at 151 °C. However, for the CR/Cu_2_O-5, the peak is in the range 135–165 °C, with the maximum temperature at 151 °C. For the CR/CuO-3, the width of the exothermic peak is smaller and is in the temperature range 128–152 °C with a maximum at 141 °C. In turn, for the CR/CuO-5, two peaks can be observed—the first at a maximum of 136 °C, and the second at 211 °C. From the obtained results, it can be observed that the type and amount of copper oxide affects not only the cross-linking of the composition but also causes changes in the structure of the entire material along with a further temperature increase in the system.

**Figure 8:**
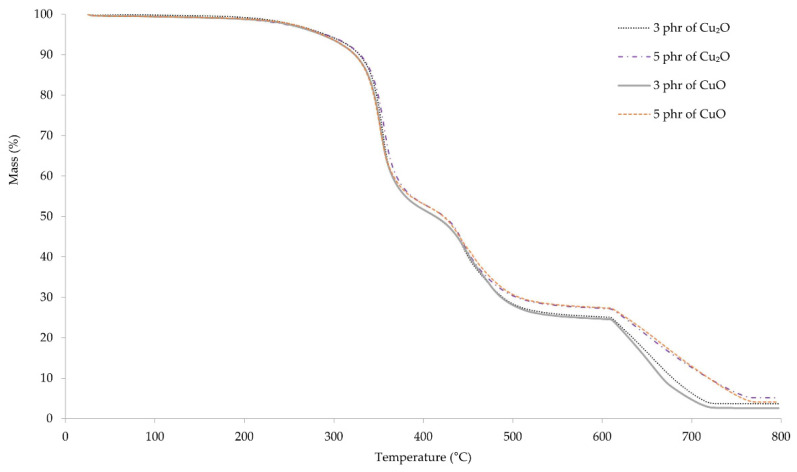
TGA spectrum of CR vulcanizates; *T* = 160 °C, *t* = 45 min.

Thermogravimetric curves allowed us to determine the processes occurring in vulcanizates during their heating. The first weight loss of the samples occurs at a temperature range 368–373 °C, where the pyrolysis of the material begins (Figure 8). The weight loss of the samples was in the range of 26–29%. The second stage of pyrolysis occurs at the temperature range of 463–466 °C. The weight loss of the samples in this stage was also about 30%. At the temperature of ~660 °C, carbon black formed during pyrolysis, combusts as a result of changing gas from argon to air. The course of the thermogravimetric curves up to the temperature of ~500 °C is almost identical. Above this temperature, it can be observed that the weight loss of samples containing 3 phr of copper oxides is greater than that of samples containing 5 phr. This may result from the lower amount of organic components that may remain unburned at higher temperatures.

## 4. Conclusions

In summary, chloroprene rubber can be cross-linked with copper oxides. The amount and type of copper oxide affect the cross-linking and properties of vulcanizates. When using copper(II) oxide, the properties are linearly dependent on the amount of CuO. The lowest cross-linking degree and mechanical properties were obtained for the CR cross-linked with 1 phr of CuO. In turn, the largest is for the CR cross-linked with 5 phr of CuO. The optimal is the use of 3 phr of CuO as CR cross-linking agent. This is evidenced by the comparable cross-linking degree and properties of vulcanizates containing 3–5 phr of CuO. In this case, an economic factor must also be included, which will promote the use of less amount of the cross-linking agent. When using Cu_2_O as a cross-linking agent, it is difficult to indicate the relationship between the amount of copper(I) oxide and the properties of vulcanizates. When analyzing individual parameters, the best results are obtained for different samples. Such lack of dependence may result from changes taking place in the substrates during the heating of the composition (e.g., oxidation of copper(I) oxide, reorganization of the network). Among the tested samples, the optimal seems to be the use of 3 phr of Cu_2_O as a cross-linking agent. This is evidenced by the high values of the tensile strength and the first elasticity constant. Infrared spectroscopy studies confirm the ability of copper oxides to cross-link chloroprene rubber. This is evidenced by the characteristic changes in the intensity of the bands. The thermal analysis allowed to determine the changes occurring in the materials during their heating. Regardless of the type and amount of copper oxide, all compositions exhibit similar characteristics, and the glass transition temperature is nearly identical. The greatest changes can be seen in the DSC analysis after exceeding the temperature of 100 °C, which may indicate structural changes in the material, depending on the cross-linking substance used. A list of abbreviations used in this article is provided in Table 5.

## Figures and Tables

**Table 1:** Tested compositions and their designations.

CR	Cu_2_O	CuO	ZnO	MgO	Stearic Acid	Symbol
100	-	-	-	-	1	CR
100	1	-	-	-	1	CR/Cu_2_O-1
100	2	-	-	-	1	CR/Cu_2_O-2
100	3	-	-	-	1	CR/Cu_2_O-3
100	4	-	-	-	1	CR/Cu_2_O-4
100	5	-	-	-	1	CR/Cu_2_O-5
100	-	1	-	-	1	CR/CuO-1
100	-	2	-	-	1	CR/CuO-2
100	-	3	-	-	1	CR/CuO-3
100	-	4	-	-	1	CR/CuO-4
100	-	5	-	-	1	CR/CuO-5
100	-	-	5	4	1	CR/ZnO/MgO

**Table 2:** Vulcametric parameters of the tested compositions determined at the temperature 160 °C.

Symbol	*t*_02_ (min)	*t*_90_ (min)	*M*_min_ (dN · m)	Δ*M*_45_ (dN · m)
CR	6.4	49.0	0.55	1.86
CR/Cu_2_O-1	3.3	38.8	0.53	2.63
CR/Cu_2_O-2	2.7	48.7	0.56	2.69
CR/Cu_2_O-3	3.1	51.0	0.59	2.78
CR/Cu_2_O-4	4.7	52.9	0.59	2.98
CR/Cu_2_O-5	7.4	53.7	0.59	3.91
CR/CuO-1	8.1	44.1	0.58	0.88
CR/CuO-2	3.4	42.3	0.58	1.19
CR/CuO-3	3.1	44.9	0.61	2.10
CR/CuO-4	1.7	38.6	0.62	2.64
CR/CuO-5	2.0	38.8	0.59	2.65
CR/ZnO/MgO	4.5	27.5	0.59	3.77

**Table 3:** Values of equilibrium swelling, elasticity constants, and real extract of CR vulcanizates; *T* = 160 °C, *t* = 45 min.

Symbol	*Q_V_*^T^ (cm^3^/cm^3^)	*Q_V_*^H^ (cm^3^/cm^3^)	*C*_1_ (kG/cm^2^)	*C*_2_ (kG/cm^2^)	*E*_R_ (mg/mg)
CR	19.26 ± 0.35	0.39 ± 0.03	0.34	2.22	0.090
CR/Cu_2_O-1	8.50 ± 0.09	0.51 ± 0.10	0.47	0.50	0.093
CR/Cu_2_O-2	10.56 ± 0.45	0.38 ± 0.01	0.64	1.09	0.102
CR/Cu_2_O-3	10.38 ± 0.39	0.40 ± 0.07	0.67	1.15	0.084
CR/Cu_2_O-4	10.22 ± 0.43	0.37 ± 0.02	0.55	1,88	0.097
CR/Cu_2_O-5	9.76 ± 0.13	0.40 ± 0.02	0.62	2.44	0.084
CR/CuO-1	8.39 ± 0.22	0.38 ± 0.02	0.38	2.53	0.090
CR/CuO-2	7.48 ± 0.40	0.42 ± 0.04	0.66	3.70	0.084
CR/CuO-3	4.70 ± 0.18	0.45 ± 0.03	0.71	2.37	0.066
CR/CuO-4	4.69 ± 0.09	0.45 ± 0.04	1.04	1.62	0.058
CR/CuO-5	4.68 ± 0.06	0.48 ± 0.02	1.32	1.36	0.057
CR/ZnO/MgO	12.24 ± 0.64	0.45 ± 0.01	0.58	0.46	0.159

**Table 4:** Mechanical properties of CR vulcanizates; *T* = 160 °C, *t* = 45 min.

Symbol	*S*_e100_ (MPa)	*S*_e200_ (MPa)	*S*_e300_ (MPa)	*TS*_b_ (MPa)	*E*_b_ (%)
CR	0.47 ± 0.01	0.52 ± 0.01	0.57 ± 0.02	1.9 ± 0.2	779 ± 15
CR/Cu_2_O-1	0.76 ± 0.06	0.97 ± 0.10	1.16 ± 0.14	6.9 ± 1.2	614 ± 21
CR/Cu_2_O-2	2.06 ± 0.10	2.44 ± 0.17	3.21 ± 0.27	12.4 ± 0.9	639 ± 14
CR/Cu_2_O-3	1.50 ± 0.19	1.73 ± 0.19	2.24 ± 0.21	13.5 ± 0.5	752 ± 40
CR/Cu_2_O-4	1.58 ± 0.06	1.83 ± 0.08	2.31 ± 0.10	13.7 ± 0.7	709 ± 12
CR/Cu_2_O-5	1.08 ± 0.02	1.28 ± 0.01	1.55 ± 0.01	10.1 ± 0.5	679 ± 23
CR/CuO-1	0.48 ± 0.01	0.63 ± 0.02	0.76 ± 0.04	6.9 ± 0.7	1092 ± 41
CR/CuO-2	0.67 ± 0.07	1.04 ± 0.14	1.46 ± 0.24	6.7 ± 2.2	822 ± 130
CR/CuO-3	0.69 ± 0.02	0.99 ± 0.01	1.27 ± 0.06	13.0 ± 1.6	962 ± 100
CR/CuO-4	0.74 ± 0.08	1.12 ± 0.16	1.53 ± 0.30	12.1 ± 1.1	917 ± 85
CR/CuO-5	0.72 ± 0.03	0.99 ± 0.04	1.20 ± 0.04	14.3 ± 1.0	1055 ± 9
CR/ZnO/MgO	0.95 ± 0.17	1.13 ± 0.19	1.33 ± 0.21	7.8 ± 1.2	680 ± 25

**Table 5:** List of abbreviations used in the article with descriptions.

Symbol	Description
IR	infrared spectroscopy
DSC	differential scanning calorimetry
TGA	thermogravimetric analysis
CR	chloroprene rubber
CSM	chlorosulfonated polyethylene
CIIR	chlorobutyl
BIIR	bromobutyl
XNBR	carboxylated acrylonitrile-butadiene rubber
SBR	styrene-butadiene rubber
BR	butadiene rubber
Cu_2_O	copper(I) oxide
CuO	copper(II) oxide
ZnO	zinc oxide
MgO	magnesium oxide
Fe_2_O_3_	iron(III) oxide
Fe_3_O_4_	iron(II,III) oxide
Al_2_O_3_	aluminum(III) oxide
CaO	calcium oxide
TiO_2_	titanium(IV) oxide
ZnCl_2_	zinc chloride
CuCl	copper(I) chloride
CuCl_2_	copper(II) chloride
phr	parts per hundred rubber
Δ*M_x_*	torque increment after a given time of heating (dN · m)
*M_x_*	torque after a given time of heating (dN · m)
*M*_min_	minimum torque (dN · m)
*t*_02_	scorch time (min)
*t*_90_	cure time (min)
*Q_V_* ^T^	equilibrium volume swelling in toluene (cm^3^/cm^3^)
*Q_V_* ^H^	equilibrium volume swelling in heptane (cm^3^/cm^3^)
*Q_W_*	equilibrium weight swelling (mg/mg)
*d*_v_	vulcanizate density (g/cm^3^)
*d*_s_	solvent density (g/cm^3^)
*m*_s_	swollen sample weight (mg)
*m*_d_	dry sample weight (mg)
*m*_d_^*^	reduced sample weight (mg)
*m*_0_	initial sample weight (mg)
*m*_m_	mineral content in the blend (mg)
*m*_t_	total weight of the blend (mg)
*m*	final sample weight (mg)
*C*_1_	first elasticity constant (kG/cm^2^)
*C*_2_	second elasticity constant (kG/cm^2^)
*P*	deformation force at λ (kG)
*λ*	deformation (λ = l/l_0_)
*l*	measuring section of the sample loaded with P (cm)
*l*_0_	measuring section of the unloaded sample (cm)
*A*_0_	cross-sectional area of the unloaded sample (cm^2^)
*E*_R_	real extract in acetone (mg/mg)
*S*_e100_	stress at elongation 100% (MPa)
*S*_e200_	stress at elongation 200% (MPa)
*S*_e300_	stress at elongation 300% (MPa)
*TS*_b_	tensile strength (MPa)
*E*_b_	elongation at break (%)
*T*_g_	glass transition temperature

## Data Availability

Data sharing not applicable.
